# Rapidly Progressive Infection of Hand After a Cat Bite

**DOI:** 10.7759/cureus.9357

**Published:** 2020-07-23

**Authors:** Tjasa Dimcic, Minja Gregoric, Vesna Breznik

**Affiliations:** 1 Department of Dermatology and Venereal Diseases, University Medical Center Maribor, Maribor, SVN; 2 Department of Plastic and Reconstructive Surgery, University Medical Center Maribor, Maribor, SVN

**Keywords:** cat-bite, hand infection, bartonella henselae

## Abstract

Cat bites represent the second most common mammalian bites after dog bites and are responsible for three-quarters of bites that result in infection. We report a case of a 60-year-old retired woman who was admitted to the surgery daily clinic due to fever and pain with three necrotic bite wounds on her hand and lymphangitis, which developed one day after she had been bitten by her cat. Prompt debridement, irrigation and drainage combined with empiric oral amoxicillin/clavulanic acid, resulted in clinical improvement and reduction of elevated inflammatory parameters specifically C-reactive protein. While wound cultures remained sterile, serology results were positive for Bartonella henselae.

## Introduction

Animal bites represent an average of 1% to 2% of patients’ visits to the ED; however, most of them are minor injuries and often go unreported [1]. Although dogs perpetrate the majority of animal bites, cat bites are responsible for three-quarters of bites that result in infection [2]. Cat bites are easily underestimated because of the minimal appearance of tissue injury [3]. Nevertheless, cats have sharp, slender teeth that penetrate deep tissues, bones, and joints and produce deep puncture wounds, inoculated with saliva and microbes, which leave a potential dead space resulting in deep infections [4]. Clinical infection after a cat bite is mostly characterized by acute onset of erythema, swelling and intense pain within 24 hours of initial injury in 70% of patients and by 48 hours in almost 90% of cases [5]. The risk factors for severe infection after a cat bite include diabetes mellitus, immunocompromised state, older age, late presentation, or suboptimal preceding treatment [6]. Sepsis, multi-organ failure and fatal outcome may ensue, if the progression of infection is not controlled [3]. In severely infected cat bites with septic shock and multi-organ failure, mortality has been reported in 25% to 60% of cases [6].

## Case presentation

**Figure 1:**
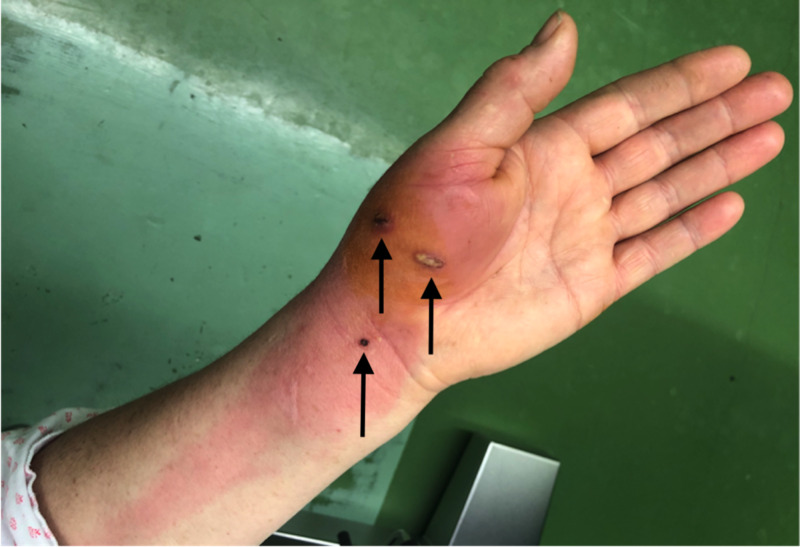
Three deep cat bite wounds surrounded by erythema and edema of right thenar muscle and wrist, propagating into a major lymphangitis on the arm

**Figure 2:**
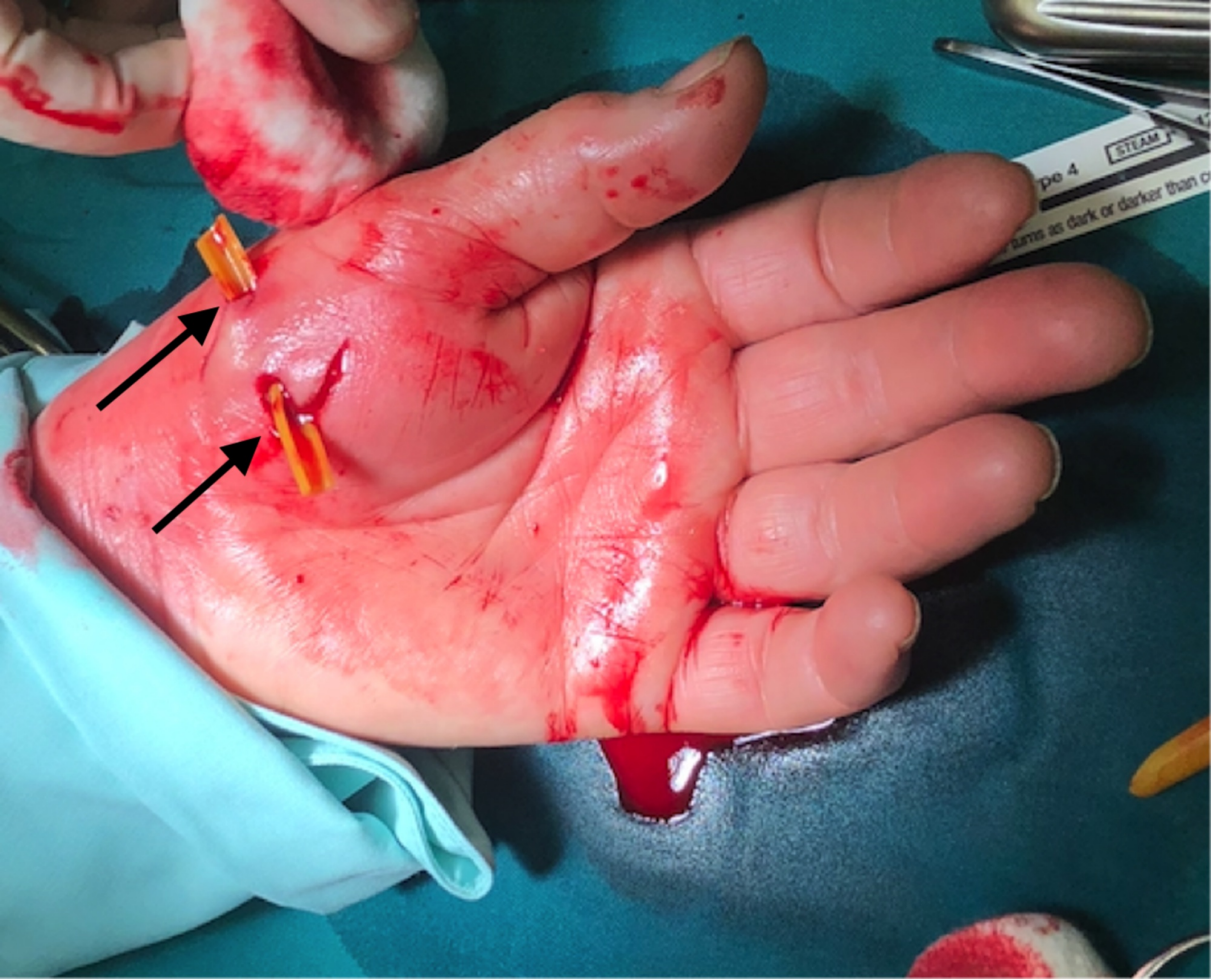
Surgical debridement, irrigation, and catheter placed into the cat bite wounds

A 60-year-old woman was bitten by her cat a day before she showed up in ED with fever (38.5 ˚C), malaise, and three deep wounds on the volar side of her right hand and wrist with prominent redness, swelling and pain, accompanied by a broad streak of redness spreading towards the elbow (Figure 1). She reported pain (visual analogue scale 8/10) and loss of sensation around the wound area. She was an active tobacco smoker for 20 pack-years. Laboratory findings revealed elevated white blood cell counts of 15.8x10^9^/L (normal: 4.0-10.0x10^9^/L) and C-reactive protein of 224.9 mg/L (normal: <3.0mg/L). Other laboratory tests such as complete blood count, heart markers, urine analysis and biochemical analysis were within reference values. Serological examination for Bartonella henselae was immediately positive (1:256). Surgical necrectomy, debridement, and drainage were performed right after the admission and oral amoxicillin/clavulanic acid treatment was initiated which rapidly acted on the patients' symptoms (Figure 2). Following surgery, the patient was hospitalised. After a third day, she was discharged clinically doing well and further followed in the outpatient clinic in one week where despite a mild pain in the bite region area she did not recall any problems also laboratory findings were normal. 

## Discussion

The hand by itself is a very vulnerable part of the human body, since it contains many superficially lying structures, such as tendons, joints, blood vessels and nerves covered by only a thin soft-tissue envelope [4]. Therefore, it is not surprising that long needle-like cats’ teeth, infected with various bacteria, can cause deep infection and rapidly spreading cellulitis and lymphangitis as in the presented patient [7]. It is reported that 45% to 65% of animal bites affect hands and wrists and frequently occur during petting or feeding the animal, as in our patient [2]. Interestingly, women are bitten by cats more frequently compared to men, which could be explained by a more affectionate attitude towards cats in women [4].

In general, the most common pathogens found in bite wounds are Pasteurella spp [8]. In cat bite wounds, they are followed by Streptococci, Staphylococci, Neisseria and Moraxella spp. Cats are also the main reservoir of Bartonella henselae, the causative agent of cat scratch disease, which was confirmed in the presented patient by serological examination. Most patients with a cat scratch disease report a history of cat scratch or bite. Whether the cat is a mostly indoor or a mostly outdoor animal probably makes a difference in the likelihood of becoming a vector in the transmission of infectious organisms [9]. The transmission of Bartonella henselae to cats is vector mediated through the cat flea, Ctenocephalides felis [10]. In patients, who demonstrate negative seroreactivity against Bartonella henselae, another Bartonella species, recently implicated in the development of cat scratch disease due to a cat bite, Bartonella clarridgeiae, should also be considered a potential causative microbe [11].

While the ideal treatment of a cat bite involves immediate radical debridement with antibiotic therapy, optimal timing and algorithm of the surgery are yet unknown [4]. The initial treatment of choice for Bartonella henselae is beta-lactam/beta-lactamase inhibitor combination, which stops bacterial growth by inhibiting the penicillin-binding proteins that are indispensable for the cross-linking process during cell wall biosynthesis. Therefore, antibiotic therapy with oral amoxicillin/clavulanic acid is considered the first-line treatment for animal bites [9]. Patients with penicillin allergy should be prescribed fluoroquinolones or trimethoprim/sulfamethoxazole. For patients older than 10 years, doxycycline can serve as an alternative [11].

Sometimes, X-rays or MRI are performed in order to detect any residual teeth from an animal bite or further detectable debris. X-ray examination is a commonly used modality, because it is inexpensive and easy to perform; however, it has low specificity in the detection of an infection, especially osteomyelitis [12]. In this case, we performed only surgical debridement and necrectomy including antibiotic therapy, clinical-laboratory follow-up. In accordance with recommendations for treatment of thenar space infections, we performed the drainage via volar and dorsal incisions, then we identified neurovascular structures, and safely excised the walls of the wounds to open the abscess cavity and minimize the bacterial load [9]. We irrigated and debrided the wound and placed a catheter into the incisions, closed the wound and placed a compressive dressing and a plaster splint.

## Conclusions

In the presented case of within hours, progressive cat bite-associated cat-scratch disease affecting the patient’s hand prompt surgical necrectomy and drainage combined with empiric amoxicillin/clavulanic acid therapy led to successful healing without any compromise to the function of the hand. Our case underlines the significance of considering surgical debridement and antibiotic therapy, since cat bites are extremely prone to infections of deeper tissues of the hand, which can result in devastating disabilities.
